# High prevalence of being Overweight and Obese HIV-infected persons, before and after 24 months on early ART in the ANRS 12136 Temprano Trial

**DOI:** 10.1186/s12981-016-0094-y

**Published:** 2016-02-25

**Authors:** Calixte Guehi, Anani Badjé, Delphine Gabillard, Eric Ouattara, Serge Olivier Koulé, Raoul Moh, Didier Ekouevi, Hugues Ahibo, Jean Baptiste N’Takpé, Gérard Kouamé Menan, Nina Deschamps, Jerôme Lecarrou, Serge Eholié, Xavier Anglaret, Christine Danel

**Affiliations:** Programme PACCI, ANRS research Site, Abidjan, Côte d’Ivoire; Unité de Soins Ambulatoire et de Conseil (USAC), 18 BP, 1125 Abidjan 18, Côte d’Ivoire; Inserm U 1219, Bordeaux University, Bordeaux, France; ISPED, Bordeaux University, Bordeaux, France; Centre de Recherche et Diagnostic sur le SIDA, (Cedres) CHU de Treichville, Abidjan, Côte d’Ivoire; Department of Infectious Diseases, Treichville Hospital, Abidjan, Côte d’Ivoire

**Keywords:** Obesity, HIV infection, Africa, Early ART

## Abstract

**Background:**

HIV is usually associated with weight loss. World health Organization (WHO) recommends early antiretroviral (ART) initiation, but data on the progression of body mass index (BMI) in participants initiating early ART in Africa are scarce.

**Methods:**

The Temprano randomized trial was conducted in Abidjan to assess the effectiveness of early ART and Isoniazid (INH) prophylaxis for tuberculosis in HIV-infected persons with high CD4 counts below 800 cells/mm^3^ without any indication for starting ART. Patients initiating early ART before December 2010 were included in this sub-study. BMI was categorized as: underweight (<18.5 kg/m^2^), normal weight (18.5–24.9 kg/m^2^), overweight (25–29.9 kg/m^2^) and obese (≥30 kg/m^2^). At baseline and after 24 months of ART, prevalence of being overweight or obese and factors associated with being overweight or obese were estimated using univariate and multivariate logistic regression.

**Results:**

At baseline, 755 participants (78 % women; median CD4 count 442/mm^3^, median baseline BMI 22 kg/m^2^) initiated ART. Among them, 19.7 % were overweight, and 7.2 % were obese at baseline. Factors associated with being overweight or obese were: female sex aOR 2.3 (95 % CI 1.4–3.7), age, aOR for 5 years 1.01 (95 % CI 1.0–1.2), high living conditions aOR 2.6 (95 % CI 1.5–4.4), High blood pressure aOR 4.3 (95 % CI 2.0–9.2), WHO stage 2vs1 aOR 0.7 (95 % CI 0.4–1.0) and Hemoglobin ≥95 g/dl aOR 3.0 (95 % CI 1.6–5.8). Among the 597 patients who attended the M24 visit, being overweight or obese increased from 20.4 to 24.8 % (p = 0.01) and 7.2 to 9.2 % (p = 0.03) respectively and factor associated with being overweight or obese was immunological response measured as an increase of CD4 cell count between M0–M24 (for +50 cells/mm^3^: aOR 1.01; 95 % CI 1.05–1.13, p = 0.01).

**Conclusion:**

The weight categories overweight and obese are highly prevalent in HIV-infected persons with high CD4 cell counts at baseline, and increased over 24 months on ART in this Sub-Saharan African population.

## Background

The HIV epidemic in Sub-Saharan Africa—the region most affected by the epidemic—has been widely documented; in 2014, this region alone accounted for 69 % of the 36.9 million people living with HIV in the world [1]. Weight loss has always been part of the natural history of HIV infection, and wasting syndrome is one of the disease severity classification criteria used by the World Health Organization (WHO) [2] and the US Centers for Disease Control and Prevention (CDC) [3]. Indeed, being underweight is a predictive factor for morbidity and mortality among HIV-infected persons [4]. Although antiretroviral treatment (ART) decreases the risk of mortality, HIV patients on ART remain at higher risk of morbidity and mortality compared to the general population [5]. In high-income countries where ART has been widely available for the past 30 years, the metabolic and cardiovascular consequences of HIV and treatment are better understood. HIV infection, independent of being overweight and obese, is recognized as a cardiovascular risk factor [6]. HIV infection and their treatment also increase the risk of diabetes [7], myocardial infarction [8], and arterial hypertension [9, 10] compared to the general population, and these cardiovascular pathologies are responsible for 7–8 % of deaths according to mortality studies conducted in France in 2005 [11].

Moreover, being overweight and obese are themselves risk factors for cardiovascular morbidity and mortality [12], and they are also often associated with hypercholesterolemia, hypertriglyceridemia, type 2 diabetes and insulin resistance [13–15]. Being overweight also increases the risk of degenerative joint such as osteoarthrosis [12]. Patients on ART experience an increase in body mass index (BMI), mainly in developed countries [16–19]. In contrast, being overweight and obese has been studied less extensively in Sub-Saharan Africa, and even less so within the HIV-infected population. WHO has issued warnings predicting the emergence of cardiovascular pathologies in resource-limited countries over the coming decades, especially due to the rise in risk factors such as being overweight and obese [20].

The recent 2010 and 2015 WHO guidelines [21–23] recommend earlier initiation of ART, but their impact in terms on being overweight and obese is unknown. We evaluated the benefits and risks of early ART (CD4 cell count below-800 and no indication to start ART according to the WHO Guidelines before 2015) was conducted in Abidjan, Côte d’Ivoire [24]. In this analysis, we describe the distribution of BMI before ART initiation and progression after 24 months of ART, as well as factors associated with varying trends, through a nested sub-study in the Temprano trial (ANRS 12136).

## Methods

### Temprano trial design

Temprano is a multi-center randomized open-label trial to assess the benefits and risks of two interventions in HIV infected participants with high CD4 cell counts: early ART initiation and 6-month isoniazid preventive therapy (IPT). [23] The trial was launched in March 2008 in nine clinical centers in Abidjan, Côte d’Ivoire, and ended in December 2014. The trial protocol was approved by the institutional review board of the French Research Agency on AIDS and viral hepatitis (ANRS, Paris) and by the Côte d’Ivoire National Ethics Committee. It has been registered on clinicaltrials.gov under the identifier NCT00495651.

The main trial inclusion criteria were: adults HIV-1 or dual HIV-1/2 infected signed informed consent; absence of ongoing active TB; CD4 count ≤800/mm^3^ and no indication to start ART immediately, according to the most recent WHO guidelines. Participants were randomized into one of four arms (i) immediate ART, (ii) deferred ART, (iii) immediate ART plus 6-month IPT, and (iv) deferred ART plus 6-month IPT. Immediate ART consisted in starting ART at enrollment irrespective of patients’ CD4 count and clinical stage. Deferred ART consisted in starting ART when WHO clinical and immunological criteria for ART initiation were met [12].The trial enrolled 2076 patients, who were followed for 30 months. The main outcome was the occurrence of a new episode of severe morbidity, including AIDS-defining diseases, non-AIDS defining severe bacterial diseases, non-AIDS defining cancers, and any event leading to death.

### Temprano trial procedures

At D0, a clinical examination including weight, height, waist size, blood pressure, and temperature was conducted, and chest radiography was prescribed. Data on socio-demographic characteristics, occupation, level of schooling, and use of tobacco and alcohol were collected using questionnaires. Blood samples were collected for analysis of blood cell count, CD4 cell count (True Count^®^ technique, FACScan^®^, Becton–Dickinson), serum transaminases, serum creatinine, serum glucose, total and LDL cholesterol, triglyceride, and plasma HIV-1 RNA (real-time PCR, Taq Man technology ABI Prism 7000, Applied Biosystems, detectability 100 copies/mL). The first-line regimen was preferably a fixed-dose combination of tenofovir disoproxil fumarate (TDF) 300 mg and emtricitabine (FTC) 250 mg (Truvada^®^, Gilead) plus efavirenz (EFV) 600 mg (Stocrin^®^, MSD). Patients with contra-indications to efavirenz were given either Truvada^®^ plus zidovudine 300 mg or Truvada® plus lopinavir/ritonavir 400/100 mg (Kaletra®, Abbott). Patients were asked to return for scheduled trial visits at Day8, M1, M2, M3, and every 3 months thereafter. Standardized questionnaires were used to record baseline and longitudinal data.

Weight was measured at each visit using the same scales and standardized procedures, with patients barefoot. The scales were calibrated regularly. BMI was defined as the weight in kilograms divided by the square of the height in meters (kg/m^2^) and classified according to the WHO as underweight (<18.5 kg/m^2^), normal (18.5–24.9 kg/m^2^), overweight (25–29.9 kg/m^2^), and obesity (30–39.9 kg/m^2^) [25].

### Participants

In this sub-study, we included all participants who initiated early ART prior to December 31, 2010. We assessed baseline characteristics according to BMI category and analyzed factors associated with being overweight or obese in univariate and multivariate analyses. We also assessed incidence of death, morbidity and loss to follow-up during the first 24 months. Severe morbidity was defined as any new AIDS-defining diseases, non-AIDS defining severe bacterial diseases, non-AIDS defining cancers, any event leading to death, and any adverse event of grade 3 or 4 on the ANRS scale (including cardiovascular, renal and neurologic events). We estimated the change in BMI between baseline and M24 in patients who survived, attended the M24 visit, and had available data. We therefore excluded the following patients: women who became pregnant, as well as patients who died, were lost to follow-up, or had missing data at the M24 visit.

### Statistical methods

Fisher exact, Chi square, and McNemar tests were used to compare patients’ baseline characteristics by BMI category. We describe changes in the prevalence of each BMI category (underweight, normal, overweight, obesity) at baseline and every 6 months until 24 months. We compare the characteristics of participants who attended the 24 months visits to those who didn’t attended this visit in an univariable analysis.

We analyse the association between being overweight or obese at baseline and the following variables: sex, age, marital status, employers status, nationality, living conditions, WHO stage, BMI, tobacco use, alcohol use, hypertension, hyperglycemia, triglyceride, cholesterol, HIV viral load, haemoglobinemia and baseline CD4 cell count (<350/mm^3^; 350–500/mm^3^; <500/mm^3^). We did the same analysis restricted to women at baseline.

Cumulative incidences of being overweight or obese at 24 months were estimated with 95 % confidence intervals among patients who were underweight or of normal weight at baseline. Logistic regression models were used to analyze the association between baseline and follow-up characteristics and being overweight or obese at months 24. We included the same variables than for the first analyse: and add follow up variables: type of ART regimen (2 NRTI + PI or 2 NRTI + NNRTI), use of IPT, and changes in CD4 cell count and viral load between M0 and M24, virological success defined as undetectable viral load at month 24 Variables with a *p* value <0.25 in univariate analysis were included in multivariate analysis. The same analysis was performed restricted to women and to people with undetectable viral load at month 24. Analyses were performed with SAS® software, version 9.2 (SAS institute Inc., Cary, North Carolina, USA).

## Results

### Baseline

Between March 2008 and December 2010, 1521 individuals infected with HIV-1 or HIV-1/2 were included in the Temprano trial. Among these, 755 (49.6 %) initiated ART immediately. Their baseline characteristics by BMI category are detailed in Table 1. Participants who attended the 24 month visit got the following antiretroviral regimen at enrollment: TDF-FTC-EFV: 443 (74 %), TDF-FTC-AZT: 59 (10 %), and TDF-FTC-LPV/r: 95 (16 %). This distribution was similar across different BMI categories (p = 0.13).

**Table 1:** Baseline characteristics of participants by baseline BMI, Temprano study, Abidjan, March 2008–December 2012 (N = 755)

Characteristics	AD	Total N = 755 (100 %)	Underweight N = 75 (10.0 %)	Normal N = 478 (63.3 %)	Overweight N = 149 (19.7 %)	Obese N = 53 (7.0 %)	P value
Women, n (%)	*755*	591 (78.3)	58 (77.3)	362 (75.7)	122 (82.0)	49 (92.4)	*0.02*
Age (years), median (IQR)	*755*	35 (29–42)	35 (29–44)	35 (29–42)	35 (30–43)	40 (33–46)	*0.01*
Electricity, n (%)	755	720 (95.4)	68 (90.7)	453 (94.8)	147 (98.7)	52 (98)	*0.006*
Refrigerator, n (%)	755	314 (41.6)	25 (33.3)	186 (39)	75 (50.3)	28 (52.8)	*0.01*
Running water, n (%)	755	628 (83.2)	57 (76)	391 (81.8)	134 (89.9)	46 (86.8)	*0.03*
Marital status, n (%)							*0.01*
Single	755	340 (45.0)	53 (33)	228 (47.7)	59 (39.6)	13 (24.5	
Married	755	315 (41.7)	24 (32)	193 (40.4)	69 (46.3)	29 (54.7)	
Divorced	755	100 (13.3)	11 (14.7)	57 (12)	21 (14)	11 (20.7)	
Employed, n (%)	*755*	510 (67.6)	38 (50.7)	326 (68.2)	105 (70.5)	41 (77.4)	*0.005*
Smokers, n (%)	751	63 (8.4)	8 (10.6)	45 (9.5)	9 (6.1)	1 (1.9)	*0.02*
Alcohol use, n (%)	754						0.11
Never		457 (60.6)	55 (73.3)	279 (58.5)	91 (61.0)	32 (60.4)	
Any use		297 (39.4)	20 (26.7)	198 (41.5)	58 (39.0)	21 (39.6)	
Not every day		290 (38.5)	19 (25.3)	192 (40.2)	58 (39.0)	21 (39.6)	
At least once a day		7 (0.9)	1 (1.4)	6 (1.3)	0 (0.0)	0 (0.0)	
WHO stage n (%)	*755*						
1		483 (64.0)	36 (48)	298 (62.3)	107 (71.8)	42 (79.3)	*10* ^*−4*^
2		190 (25.2)	27 (36)	119 (25.0)	33 (22.2)	11 (20.7)	
3		77 (10.2)	10 (13.3)	58 (12.1)	9 (6.0)	0 (0.0)	
4		5 (0.6)	2 (2.7)	3 (0.6)	0 (0.0)	0 (0.0)	
HTN, n (%)	755	37 (4.9)	1 (1.3)	14 (2.9)	10 (6.7)	12 (22.6)	*10* ^*−4*^
BMI median (IQR)	755	22.3 (20–25.2)	17.6 (16.6–18.1)	21.5 (20.2–23.0)	26.5 (25.6–27.7)	31.8 (30.8–35.0)	
Reported weight loss, n (%)	755						*0.004*
Never		645 (85.4)	60 (80.0)	400 (83.7)	133 (89.3)	52 (98.0)	
<10 %		58 (7.7)	12 (16.0)	36 (7.5)	9 (6.0)	1 (2.0)	
>10 %		52 (6.9)	3 (4.0)	42 (8.8)	7 (4.7)	0 (0.0)	
CD4 (/mm^3^), median (IQR)	755	442 (348–541)	446 (336–536)	439 (342–541)	440 (349–545)	481 (432–534)	0.37
CD4 count (/mm^3^), n (%)							
≤350		201 (26.6)	19 (25.3)	134 (28.0)	40 (26.8)	8 (15.1)	0.62
350–500		300 (39.7)	31(41.3)	187 (39.1)	59 (39.6)	23 (43.4)	
>500		254 (33.7)	25 (33.4)	157 (32.9)	50 (33.6)	22 (41.5)	
Viral load (log), median (IQR)	754	4.7 (3.98-5.26)	4.84 (4.07–5.60)	4.72 (3.97–5.28)	4.67 (4.07–5.17)	4.44 (3.89–4.98)	0.11
Hemoglobin <95 g/dL, n (%)	754	101 (13.4)	19 (25.3)	69 (14.5)	12 (8.0)	1 (2.0)	*10* ^*−4*^
Hyperglycemia, n (%)	752	4 (0.5)	0 (0.0)	3 (0.6)	0 (0.0)	1 (1.9)	0.40
LDL^a^ >4.13 mmol/L, n (%)	755	93 (12.3)	8 (10.7)	53 (11.1)	18 (12.1)	14 (26.4)	*0.01*
HyperTG, n (%)	754	13 (1.7)	1 (1.3)	7 (1.5)	3 (2.0)	2 (3.8)	*0.26*
Abdominal obesity^b^, n (%)	755	128 (17.0)	0 (0.0)	26 (5.4)	57 (38.2)	45 (85.0)	*10* ^*−4*^
Metabolic syndrome^c^, n (%)	755	47 (6.2)	0 (0)	11 (23.4)	16 (34)	20 (42.6)	*10* ^*−4*^

Italic values are statistically significant

*AD* available data, *N, n* Number, *%* percentage, *IQR* interquartile range, *P value* comparisons are made using the chi square or fisher test to compare proportions or then the Wilcoxon test for median comparison, *BMI* body mass index, underweight BMI <18.5 kg/m^2^, *Normal* BMI 18.5–24.9 kg/m^2^, *Overweight* BMI 25–29.9 kg/m^2^, *Obese* BMI >30 kg/m^2^, *Smokers* report smoking regularly and at least one pack of cigarettes daily, *Employed* report holding a job in the public, private, or informal sector, *SBP* systolic blood pressure, *DBP* diastolic blood pressure, *HTN* arterial hypertension (SBP ≥140 mmHg and DBP ≥90 mmHg), *HyperTG* hypertriglyceridemia (>2.3 mmol/L), *Hyperglycemia* blood glucose ≥7 mmol/L

^a^ LDL estimated with Friedwald formula: (Total Cholesterol)—(HDL Cholesterol)—(TG/5)

^b^ Abdominal obesity (>101 cm for men; >87 cm for women)

^c^ Metabolic syndrome: At least three of the five following criteria: hyperglycemia (≥7 mmol/L); low HDL (<1 mmol/L in men or <1.3 mmol/L in women); hypertriglyceridemia (>1.7 mmol/L); abdominal obesity (>101 cm for men; >87 cm for women) and HTN (SBP >140 mmHg or DBP >90 mmHg)

### Follow-up

At 24 months of follow-up, among the 755 participants who initiated ART, 13 (1.7 %) had died, 16 (2.1 %) were lost-to-follow up, 68 (9.0 %) women became pregnant, and 61 (6.7 %) patients were missing weight data at M24; thus, 597 (79.1 %) participants remained at M24. Of these 597 three quarters were women (75.0 %), their median age 36 years (Interquartile range [IQR] 31–44), and median BMI 22.5 kg/m^2^ (IQR 20.2–25.3); their median CD4 cell count and viral load were 439 cells/mm^3^ (IQR 342–535) and 4.6 log_10_/mL (IQR 3.9–5.2) respectively. Compared to the 158 participants who didn’t attended the 24 months visit, participants who attended the 24 months visit were less often female (75 vs 90 % p = 0.0001), older(median age 37 years old (IQR 31–44) vs 31 years old (IQR 26–36) p = 0.0001), with a lower viral load [4.6 log10/ml (IQR 3.9–5.2) vs 4.8 log10/ml (IQR 4.1–5.5) p = 0.01].

Before ART initiation, hormonal contraception was prescribed for 33 (5.8 %) women. Occurrence of pregnancy during follow-up was also comparable across groups (underweight: n = 6/58, 10.3 %; normal weight: n = 47/362, 13.0 %; overweight: n = 11/122, 9.0 %; obese: n = 4/49, 8.2 %; p = 0.54).

Missing weight data at M24 was equally distributed across BMI categories. Cumulative incidences by baseline BMI category for loss to follow-up, death, and severe morbidity are provided for the 597 participants in Table 2. Of the 13 who died, 11 (84.6 %) were women. The proportion of deaths at 24 months was higher in the underweight category (6.7 %) compared to all others (p = 0.04).

**Table 2:** Participants characteristics at 24 months of follow-up by baseline BMI, Temprano study, Abidjan, March 2008–December 2012 (N = 755)

	Baseline BMI				
	Underweight	Normal	Overweight	Obese	*P* value
	N = 75 (10.0 %)	N = 478 (63.3 %)	N = 149 (19.7 %)	N = 53 (7.0 %)	
Vital status at month 24 (N = 755)					
Loss to follow-up	0 (0.0)	12 (2.5)	3 (2.0)	1 (1.9)	0.67
Death	5 (6.7)	5 (1.1)	2 (1.3)	1 (1.9)	*0.04*
Morbidity*	7 (9.3)	26 (5.4)	5 (3.4)	3 (5.7)	0.19
Endpoints (N = 597)**					
At least one modification of ART***	14 (18.7)	81 (17.0)	21 (14.1)	5 (9.4)	0.42
∆CD4 (M24-M0)	225 (101–333)	231 (111–393)	231 (121–375)	214 (93–384)	0.80
VL <300 cp/ml at M24	39 (73.6)	294 (82.6)	92 (80.7)	34 (87.2)	0.33
Hemoglobin <95 g/dL at M24	3 (5.8)	15 (4.3)	2 (1.8)	1 (2.5)	0.17

Italic value are statistically significant

*N* number, *BMI* body mass index, *Underweight* BMI <18.5 kg/m^2^, *Normal* BMI 18.5–24.9 kg/m^2^, *Overweight* BMI 25–29.9 kg/m^2^, *Obese* BMI >30 kg/m^2^, *P value* comparisons are made using the chi square or fisher test to compare proportions or then the Wilcoxon test for median comparison

* Morbidity: any WHO stage 3 or 4 clinical event or any adverse event of grade 3 or 4 on the ANRS scale

** Endpoints for the 597 patients who completed 24 months of antiretroviral treatment

*** ART: antiretroviral therapy

**Fig. 1:**
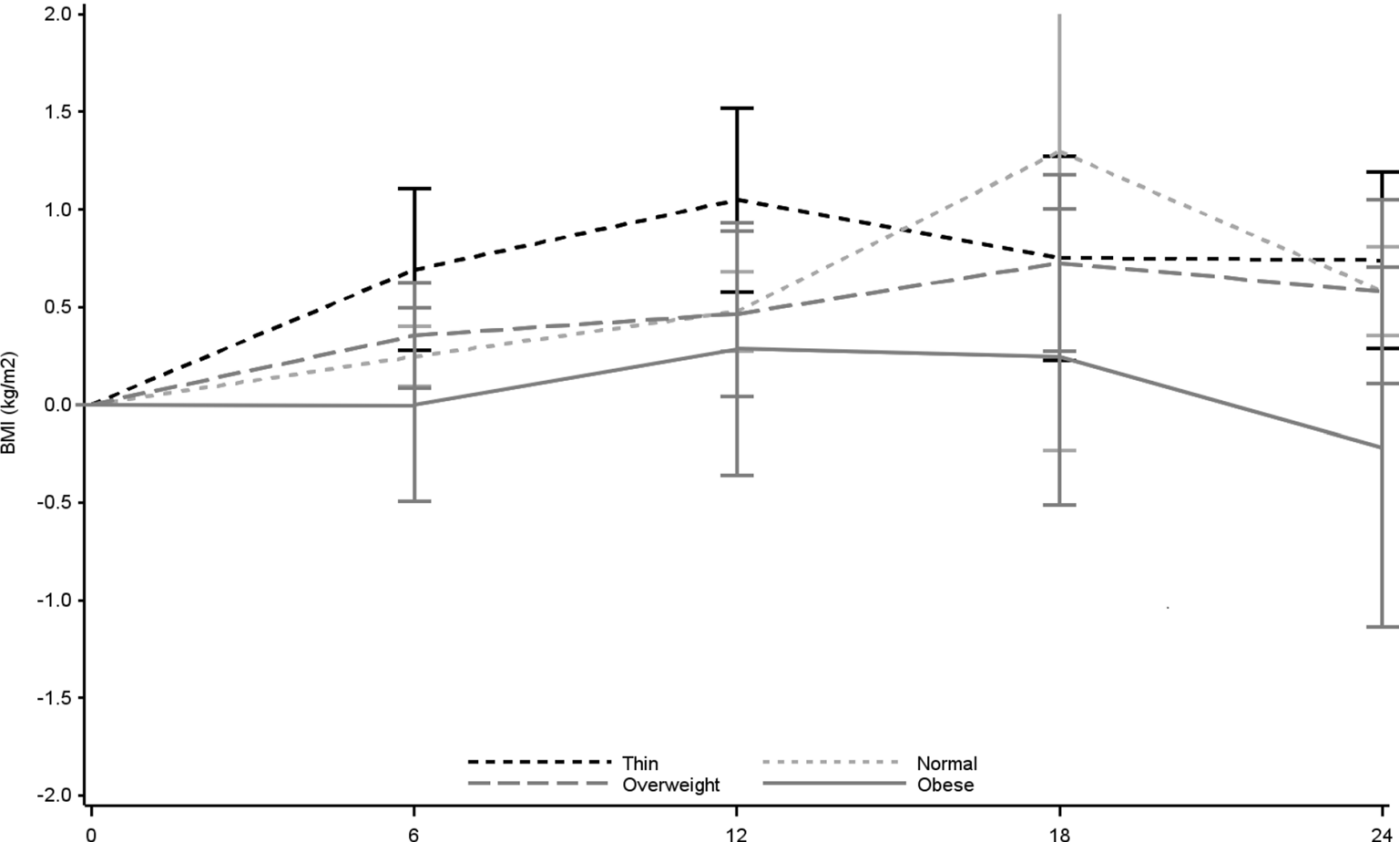
Changes of BMI vs baseline (Mean and 95 % confidence intervals) according to initial BMI category, among 597 patients who attended the 24-month visit in the Temprano trial. *N* number, *%* percentage, BMI body mass index, underweight BMI <18.5 kg/m^2^, normal BMI 18.5–24.9 kg/m^2^, Overweight BMI 25–29.9 kg/m^2^, Obese BMI >30 kg/m^2^

Figure 1 presents changes in BMI during the 24 months of follow up on ART. Median changes in BMI between M0 and M24 were 0.53 kg/m^2^ (IQR −0.35, 1.62) among those who were initially underweight, 0.39 kg/m^2^ (IQR −0.75, 1.77) among those with normal-weight patients, 0.42 kg/m^2^ (IQR −1.11, 1.77) among those with overweight, and 0.37 kg/m^2^ (IQR −1.64, 1.56) among those obese.

After 24 months of follow-up, 24/58 (41.4 %) of patients who were underweight at baseline switched to a higher BMI class, and 10/43 (23.2 %) of those who were obese at baseline lost weight and reached a lower category (Table 3). There were 19 (5.1 %) who were initially of normal weight and became underweight, while 59 (15.8 %) went from normal weight to being overweight or obese. Among those initially overweight, an equal number (15.6 %) switched to the normal weight and obese BMI categories. In total, 149 (25.0 %) participants experienced a change in BMI category during follow-up. The proportion of obese and overweight persons increased significantly, from 7.2 to 9.2 % (p = 0.03), and from 20.4 to 24.8 % (p = 0.01) respectively, whereas the proportion of those with normal weight decreased from 62.6 to 57.1 % (p = 0.002).

**Table 3:** Changes in BMI category after 24 months of follow-up compared to initial BMI category, Temprano study, Abidjan, March 2008–December 2012 (N = 597)

	BMI category after 24 months of ART (n = 597)				
	Underweight	Normal	Overweight	Obese	Total
	N (%)	N (%)	N (%)	N (%)	N (%)
Initial BMI category					
Underweight	34 (58.6)	24 (41.4)	0 (0.0)	0 (0.0)	58 (9.7)
Normal	19 (5.1)	296 (79.1)	56 (15.0)	3 (0.8)	374 (62.7)
Overweight	0 (0.0)	19 (15.6)	84 (68.8)	19 (15.6)	122 (20.4)
Obese	0 (0.0)	2 (4.7)	8 (18.6)	33 (76.7)	43 (7.2)
Total	53 (8.9)	341 (57.1)	148 (24.8)	55 (9.2)	597 (100.0)

*N* number,  *%* percentage, *BMI* body mass index, *Underweight* BMI <18.5 kg/m^2^, *Normal* BMI 18.5–24.9 kg/m^2^, *Overweight* BMI 25–29.9 kg/m^2^, *Obese* BMI >30 kg/m^2^

The prevalence of being overweight or obese increased significantly among the 448 women, from 30 % at baseline to 38 % at M24 (p = 10^−4^), but remained unchanged among the 149 men (20 to 22 %; p = 0.46). The proportion of women with abdominal obesity increased significantly, from 107 (24 %) to 135 (30 %) at M24 (p = 0.002). No men had abdominal obesity either at baseline or at M24.

### Factors associated with being overweight or obese

Factors associated with being overweight or obese were analyzed at baseline without ART, and at 24 months on ART. In a multivariable analysis, factors associated with being overweight or obese at baseline (N = 755) were: female sex, being older, best living conditions, WHO stage 1, high blood pressure and hemoglobin (Hb) >9.5 g/dL (Table 4). In a multivariable including only women (n = 591), there was a significant association between being overweight or obese and the following factors: Higher living conditions (Moderate vs Bad aOR 1.9; (95 % CI 1.0–3.5), High vs Bad aOR 2.7 (95 % CI 1.5–5,0); p = 0.003) hypertension (aOR 9.0; (95 % CI 3.4–24.0) p < 0.001), Hemoglobin ≥9.5 g/dL [aOR 3.2; (95 % CI 1.6–6.2), p = 0.0007], (Appendix Table 5).

**Table 4:** Association between baseline characteristics and being overweight or obese in the Temprano trial, Abidjan, March 2008–December 2012 (N = 755)

Variables	Unit	Univariable analysis			Multivariable analysis		
		OR	95 % CI	P value	aOR	95 % CI	P value
Sex	Women/men	1.74	1.13–2.68	*0.01*	2.33	1.46–3.72	*0.0004*
Age	/5 years	1.10	1.05–1.15	*0.03*	1.14	1.08–1.20	*0.006*
Employed	Yes/no	1.35	0.94–1.93	*0.09*	–	–	–
Nationality	Ivoirian/non-ivoirian	1.45	0.87–2.42	*0.15*	–	–	–
Marital status	Married/single	1.68	1.18–2.39	*0.007*	–	–	–
	Divorced/single	1.75	1.07–2.87		–	–	
Living conditions	Moderate vs bad	1.56	0.93–2.61	*0.001*	1.76	1.03–3.01	*0.001*
	Best vs bad	2.37	1.42–3.95		2.61	1.53–4.45	
Smoker	Yes/no	0.49	0.24–0.98	*0.03*	–	–	–
Alcohol use	Yes/no	0.98	0.70–1.36	0.92	–	–	–
WHO stage	2 vs 1	0.67	0.45-0.99	*0.0007*	0.71	0.47–1.07	*0.006*
	3 vs 1	0.27	0.13–0.56		0.32	0.15–0.68	
HBP (≥140/90 mmHg)	Yes/no	4.38	2.22–8.63	*0.0001*	4.36	2.06–9.20	*0.0001*
CD4 count	350–500 vs ≤350	1.19	0.79–1.81	0.54	–	–	–
	>500 vs ≤350/mm^3^	1.26	0.82-1.92		–	–	–
Viral load	/1 log	0.90	0.79–1.04	*0.16*	–	–	–
Hemoglobin ≥95 g/dL	Yes/no	2.75	1.50–5.05	*0.001*	3.09	1.63–5.86	*0.0005*
HyperTG	Yes/no	1.72	0.55–5.34	*0.34*			
LDL >4.13 mmol/L	Yes/no	1.51	0.95–2.41	*0.07*	–	–	–
Hyperglycemia	Yes/no	0.91	0.09–8.82	0.93	–	–	–

Italic values are statistically significant

*OR* odds ratio, *aOR* adjusted odds ratio, *95* *% CI* 95 % confidence interval, *TG* triglycerides, *LDL* low-density lipoprotein, *HBP* high blood pressure (systolic blood pressure ≥140 mmHg and diastolic blood pressure ≥90 mmHg), *HyperTG* Hypertriglyceridemia >2.3 mmol/L, *Hyperglycemia* blood glucose ≥7 mmol/L, Living condition is a composite variable that we created using the variables: water, electricity and fridge. The conditions were: *Bad* presence of only running water in the patient’s home, *Moderate* presence of water and electricity, *Best* presence of water, electricity and fridge, *P value* comparisons are made using the chi square or fisher test to compare proportions or then the Wilcoxon test for median comparison

**Table 5:** Association between baseline characteristics and being overweight or obese at baseline in the Temprano trial, Abidjan, March 2008–December 2012: results of univariate and multivariate analyses, restricted to women (N = 591)

Variables	Unit	Univariate analysis			Multivariate analysis		
		OR	95 % CI	P value	aOR	95 % CI	P value
Age	/5 years	1.01	0.99–1.03	*0.14*	–	–	–
Employed	Yes/no	1.45	0.99–2.12	*0.05*	–	–	–
Nationality	Ivoirian/non-ivoirian	1.33	0.75–2.36	0.32	–	–	–
Marital status	Married/single	1.64	1.10–2.42	*0.03*	–	–	–
	Divorced/single	1.47	0.87–2.46		–	–	
Living conditions	Moderate vs bad	1.74	0.98–3.11	*0.002*	1.91	1.04–3.51	*0.003*
	Best vs bad	2.61	1.47–4.65		2.74	1.50–5.01	
Smoker	Yes/no	1.65	0.46–5.94	0.43	–	–	–
Alcohol use	Yes/no	1.09	0.75–1.58	0.63	–	–	–
Contraceptive	Yes/no	0.93	0.46–1.86	0.86	–	–	–
WHO stage	2 vs 1	0.69	0.45–1.06	*0.007*	0.76	0.49–1.20	*0.03*
	3 vs 1	0.32	0.14–0.70		0.37	0.17–0.82	
HBP(≥140/90 mmHg)	Yes/no	8.62	3.38–22.00	*0.0001*	9.06	3.42–24.02	*0.0001*
CD4 count	350–500 vs ≤350	1.17	0.74-1.85	0.41	–	–	–
	>500 vs ≤350/mm^3^	1.37	0.85–2.20		–	–	–
Viral load	/1 log10 copies/ml	0.90	0.77–1.04	*0.16*	–	–	–
Hemoglobin ≥95 g/dL	Yes/no	3.12	1.65–5.90	*0.0004*	3.20	1.63–6.28	*0.0007*
HyperTG	Yes/no	3.32	0.73–15.00	*0.11*	–	–	–
LDL >4.13 mmol/L	Yes/no	1.67	1.00–2.79	*0.04*	–	–	–
Hyperglycemia	Yes/no	1.22	0.11–13.64	0.86	–	–	–

Italic values are statistically significant

*OR* odds ratio, *aOR* adjusted odds ratio, *95* *% CI* 95 % confidence interval, *TG* triglycerides, *LDL* low-density lipoprotein, *HBP* high blood pressure (systolic blood pressure ≥140 mmHg and diastolic blood pressure ≥90 mmHg), *HyperTG* hypertriglyceridemia >2.3 mmol/L, hyperglycemia Blood glucose ≥7 mmol/L, IPT isoniazid preventive therapy, *ART* antiretroviral therapy, *P value* comparisons are made using the chi square or fisher test to compare proportions or then the Wilcoxon test for median comparison

Living condition is a composite variable that we created using the variables: water, electricity and fridge. The conditions were: *Bad* presence of only running water in the patient’s home, *Moderate* presence of water and electricity, *Best* presence of water, electricity and fridge

In a multivariable analysis at 24 months, the only factor significantly associated with being overweight or obese among the 432 patients who were not overweight or obese at baseline were: immunological response as measured as an increase of CD4 cell count between M0 to M24 (for +50 cells/mm^3^: aOR 1.09; 95 % CI 1.02–1.17, p = 0.01). (Appendix Table 6). The results was similar for the analysis restricted to the women (N = 313) (Appendix Table 7) and the analysis for the persons with undetectable viral load at 24 months (N = 333) (Appendix Table 8).

**Table 6:** Association between baseline and follow up characteristics with being overweight or obese at 24 months for non obese or overweight participants at baseline, in the Temprano trial, Abidjan, March 2008–December 2012: results of univariate and multivariate analysis (N = 432)

Variables	Unit	Univariate analysis			Multivariate analysis		
		OR	95 % CI	P value	aOR	95 % CI	P value
Sex	Women/men	1.92	0.93–3.94	*0.07*	1.53	0.65–3.58	0.32
Age	/1 year	1.01	0.98–1.04	0.29	–	–	–
Employed	Yes/no	1.52	0.81–2.84	*0.18*	1.72	0.88–3.38	0.11
Nationality	Ivoirian/non-ivoirian	1.40	0.55–3.54	0.47	–	–	–
Marital status	Married/single	1.28	0.69–2.38	0.31	–	–	–
	Divorced/single	1.84	0.83–4.06		–	–	
Living conditions	Moderate vs bad	1.33	0.57–3.08	0.64	–	–	–
	Best vs bad	1.50	0.63–3.52		–	–	
Smoker	Yes/no	0.43	0.13–1.45	*0.17*	0.52	0.13–1.98	0.34
Alcohol use	Yes/no	0.65	0.36–1.19	*0.17*	0.71	0.37–1.36	0.31
WHO stage	2 vs 1	1.15	0.61–2.15	0.35	–	–	–
	3 vs 1	0.50	0.17–1.47		–	–	
Viral load at baseline	/1 log10 copies/ml	1.35	1.01–1.79	*0.03*	1.25	0.92–1.69	0.14
∆CD4	/50 CD4/mm^3^	1.11	1.04–1.18	*0.001*	1.09	1.02–1.17	*0.01*
Hemoglobin ≥95 g/dL	Yes/no	0.59	0.30–1.17	*0.13*	0.67	0.32–1.42	0.30
IPT	Yes/no	0.95	0.54–1.67	*0.88*	–	–	–
ART regimen	INNRTI vs PI	1.28	0.55–2.96	*0.56*	–	–	–

Italic values are statistically significant

*OR* odds ratio, *aOR* adjusted odds ratio, *95* *% CI* 95 % confidence interval, *TG* triglycerides, *LDL* low-density lipoprotein, *HBP* high blood pressure (systolic blood pressure ≥140 mmHg and diastolic blood pressure ≥90 mmHg), *HyperTG* hypertriglyceridemia >2.3 mmol/L, hyperglycemia Blood glucose ≥7 mmol/L, IPT isoniazid preventive therapy, *ART* antiretroviral therapy, *P value* comparisons are made using the chi square or fisher test to compare proportions or then the Wilcoxon test for median comparison

Living condition is a composite variable that we created using the variables: water, electricity and fridge. The conditions were: *Bad* presence of only running water in the patient’s home, *Moderate* presence of water and electricity, *Best* presence of water, electricity and fridge

**Table 7:** Association between baseline and follow up characteristics with being overweight or obesity at 24 months for non obese or overweight women at baseline, in the Temprano trial, Abidjan, March 2008–December 2012: results of univariate and multivariate analysis, (N = 313)

Variables	Unit	Univariate analysis			Multivariate analysis		
		OR	95 % CI	P value	aOR	95 % CI	P value
Age	/1 year	1.01	0.98–1.05	*0.24*	1.01	0.98–1.05	0.36
Employed	Yes/no	1.68	0.86–3.30	*0.12*	1.75	0.86–3.59	0.12
Nationality	Ivoirian/non-ivoirian	1.80	0.68–4.75	*0.23*	1.76	0.64–4.85	0.27
Marital status	Married/single	1.23	0.60–2.51	0.37	–	–	–
	Divorced/single	1.78	0.79–4.01		–	–	
Living conditions	Moderate vs bad	1.38	0.55–3.43	0.77	–	–	–
	Best vs bad	1.31	0.51–3.38		–	–	
Smoker	Yes/no	1.12	0.12–9.86	0.91	–	–	–
Alcohol use	Yes/no	0.61	0.30–1.24	*0.17*	0.59	0.27–1.27	0.17
WHO stage	2 vs 1	1.02	0.50–2.08	0.29	–	–	–
	3 vs 1	0.31	0.07–1.38		–	–	
∆CD4	/50 CD4/mm^3^	1.09	1.02–1.17	*0.01*	1.09	1.01–1.17	*0.03*
Viral load at baseline	/1 log10 copies/ml	0.90	0.79–1.04	*0.16*	1.25	0.90–1.75	0.18
Hemoglobin ≥95 g/dL	Yes/no	0.57	0.28–1.17	0.43	0.62	0.29–1.35	0.23
Virologic success at M24	Yes/no	1.44	0.57–3.61	0.43	–	–	–
IPT	Yes/no	0.95	0.54–1.67	0.88	–	–	–
ART regimen	INNRTI vs PI	1.28	0.55–2.96	0.56	–	–	–

Italic values are statistically significant

*OR* odds ratio, *aOR* adjusted odds ratio, *95* *% CI* 95 % confidence interval, *TG* triglycerides, *LDL* low-density lipoprotein, *HBP* high blood pressure (systolic blood pressure ≥140 mmHg and diastolic blood pressure ≥90 mmHg), *HyperTG* hypertriglyceridemia >2.3 mmol/L, hyperglycemia Blood glucose ≥7 mmol/L, IPT isoniazid preventive therapy, *ART* antiretroviral therapy, *P value* comparisons are made using the chi square or fisher test to compare proportions or then the Wilcoxon test for median comparison

Living condition is a composite variable that we created using the variables: water, electricity and fridge. The conditions were: *Bad* presence of only running water in the patient’s home, *Moderate* presence of water and electricity, *Best* presence of water, electricity and fridge

**Table 8:** Association between participants characteristics (not overweight and obesity at baseline) and overweight and obesity at 24 months in the Temprano trial, Abidjan, March 2008–December 2012: sensibility analysis in patients with undetectable viral load at M24 (N = 333)

Variables	Unit	Univariate analysis			Multivariate analysis		
		OR	95 % CI	P value	aOR	95 % CI	P value
Sex	Women/men	1.86	0.83–4.17	*0.12*	1.21	0.48–3.07	0.67
Age	/1 year	1.02	0.99–1.06	*0.13*	1.02	0.99–1.06	0.11
Employed	Yes/no	1.33	0.66–2.70	0.41	–	–	–
Nationality	Ivoirian/non-ivoirian	1.20	0.39–3.68	0.74	–	–	–
Marital status	Married/single	1.30	0.64–2.62	0.25	–	–	–
	Divorced/single	2.07	0.87–4.90		–	–	
Living conditions	Moderate vs bad	1.12	0.42–2.99	0.57	–	–	–
	Best vs bad	1.52	0.57–4.01		–	–	
Smoker	Yes/no	0.40	0.09–1.74	0.22	0.40	0.08–1.99	0.26
Alcohol use	Yes/no	0.63	0.32–1.22	*0.17*	0.70	0.34–1.40	0.31
WHO stage	2 vs 1	1.14	0.56–2.31	0.47	–	–	–
	3 vs 1	0.50	0.14–1.74		–	–	
∆CD4	/50 CD4/mm^3^	1.11	1.03–1.19	*0.005*	1.12	1.04–1.21	*0.003*
Hemoglobin ≥95 g/dL	Yes/no	0.44	0.21–0.93	*0.03*	0.46	0.20–1.01	0.05
IPT	Yes/no	0.98	0.52–1.84	*0.96*	–	–	–
ART regimen	INNRTI vs PI	1.25	0.46–3.36	*0.65*	–	–	–

Italic values are statistically significant

*OR* odds ratio, *aOR* adjusted odds ratio, *95* *% CI* 95 % confidence interval, *TG* triglycerides, *LDL* low-density lipoprotein, *HBP* high blood pressure (systolic blood pressure ≥140 mmHg and diastolic blood pressure ≥90 mmHg), *HyperTG* hypertriglyceridemia >2.3 mmol/L, hyperglycemia Blood glucose ≥7 mmol/L, IPT isoniazid preventive therapy, *ART* antiretroviral therapy, *P value* comparisons are made using the chi square or fisher test to compare proportions or then the Wilcoxon test for median comparison

Living condition is a composite variable that we created using the variables: water, electricity and fridge. The conditions were: *Bad* presence of only running water in the patient’s home, *Moderate* presence of water and electricity, *Best* presence of water, electricity and fridge

## Discussion

The prevalence of being overweight or obese in our study population was 27 % at ART initiation and slightly higher (30 %) among women. The prevalence in women was similar that reported in the general population of women in Abidjan in 2010 (25.8 %) [26]. The percentage participants of overweight or obese increased significantly to 32 % after 24 months of ART (38 % among women). As noted previously, people who were already overweight were at greater risk of becoming obese [19]. People living with HIV typically present with low CD4 counts and low BMI when initiating ART [27–29]. This differs from our study population, which was selected to start early antiretroviral treatment with CD4 counts below 800/mm^3^ and no criteria for starting ART according to WHO guidelines (inclusion criteria in the trial) Previous studies have evoked racial origin as a risk factor for overweight and obesity [30], but living conditions, type of diet [31, 32], and economic and sociocultural factors have been shown to play an important role in the development of obesity [33, 34]. Moreover, our population is mainly urban, which implies a certain standard of living compared to rural populations, as well as a more sedentary life style [35] and we confirm the role of economic incomes as a role in being overweight or obese in our study.

The proportion of women in our study was very high (75 %), and they have been reported to be more likely at risk of being overweight or obese [36]. Genetic and hormonal factors that may affect BMI may play a role in weight gain [37–40], but there are other factors such as previous pregnancy which we were unable to assess due to lack of data on past obstetric history of these women. Another explanation could have been use of oral contraceptive, but in our population study, only 5,3 % of women had an oral contraceptive (very close to the general population in Côte d’Ivoire [35]; and a recent review showed that hormonal contraceptive is not really proved to be a factor affecting weight gain [41].

In contrast to more immune-compromised populations [30], we did not observe any association between BMI and initial CD4 cell counts, which is unusual [16] probably due to the homogeneity of our study population. The type of ART regimen was not associated with being or becoming overweight or obese, as it was in other studies with association with protease inhibitors [16, 19].

Among patients initiating early antiretroviral treatment, our study showed that there is a relationship between an increase in CD4 cell count between baseline and 24 months and becoming overweight or obese. Other studies have also noted an association between weight gain and baseline CD4 level [42] Without ruling out other factors, we guess that this is due to immune reconstitution that prevents the occurrence of opportunistic infections which can alter the general condition of patients; the absence of opportunistic infections in turn promotes the general well-being of patients, including weight gain. But, the issue of whether weight gain promotes CD4 recovery (e.g., through the effects of adipokines such as leptin) or whether more robust CD4 recovery is a sign of a greater decline of uncontrolled HIV viremia which then leads to weight gain is an unresolved issue. Regardless of the factors underlying obesity, the prevalence in our population highlights its importance as a cardiovascular risk factor and should be addressed. In our study, at enrollment, there was a statistically significant association between being overweight or obese and other known cardiovascular risk factors such as age, [16], and hypertension [17, 39]. HIV infection in obese persons seems to mainly worsens cardiovascular parameters, and not metabolic [43]. However, being overweight or obese is an independent cardiovascular risk factor. Previous publications have highlighted the protective effect of being overweight compared to normal weight, but the risk of all-cause mortality is significantly higher with up to a three-fold risk among obese persons [44]. An increased risk of death from cardiovascular causes has been reported in patients who are overweight [45] and in those with abdominal obesity [46–49].

After 24 months of follow-up, low BMI was associated with morbidity and mortality in our participants. The major of cause of morbidity and mortality was infection, especially tuberculosis and invasive bacterial diseases, which occur at increasing rates with diminishing immunity [5]. Immune reconstitution during ART decreases the incidence of morbidity and mortality, but rates remain high in the first months of antiretroviral therapy [24, 50]. With the significant increase in becoming overweight or obese that was observed at only 24 months of ART, it can be postulated that after a few years, the risk of morbidity and mortality linked to becoming overweight or obese could increase. Prevention will increase awareness of this risk in a population where thinness is stigmatized as being associated with illness, and being overweight is admired not only as a criterion of beauty but also of social well-being [51, 52]. The risks associated with being overweight are thus rarely taken into account by patients and physicians.

The limitations of this study were as follows. We did not collect data on dietary habits or physical exercise, or obstetric as previously noted, important data on this outcome. As well, asking participants to fast before coming at hospital in case of sampling is an usual recommendation, but we have no proof that they were really fasting.

## Conclusion

Antiretroviral drugs have improved the prognosis of people living with HIV and initiation of ART is recommended increasingly earlier to limit the risks of morbidity and mortality even at high CD4 counts [22]. However, our study shows that the risk of being overweight or obese is high in this population, especially in women. Notably women are the most HIV-vulnerable population in Sub-Saharan Africa, accounting for 58 % of all people living with HIV [1]. Being overweight or obese should be taken into account in prevention policies in the years to come, both in persons living with HIV as well as in the general population.

Presented in part at the 7e Conférence Francophone VIH/SIDA, Montpellier, 27–30 April 2014, Abstract N°S025.2.

## Appendix

See Tables 5, 6, 7 and 8.
